# A New Carbon Allotrope with Six-Fold Helical Chains in all-*sp*^2^ Bonding Networks

**DOI:** 10.1038/srep04339

**Published:** 2014-03-11

**Authors:** Jian-Tao Wang, Changfeng Chen, Enge Wang, Yoshiyuki Kawazoe

**Affiliations:** 1 Beijing National Laboratory for Condensed Matter Physics, Institute of Physics, Chinese Academy of Sciences, Beijing 100190, China; 2 Department of Physics and High Pressure Science and Engineering Center, University of Nevada, Las Vegas, Nevada 89154, USA; 3 International Center for Quantum Materials, School of Physics, Peking University, and Collaboration Innovation Center of Quantum Matter, Beijing 100871, China; 4 New Industry Creation Hatchery Center, Tohoku University, Sendai 980-8579, Japan; 5 Institute of Thermophysics, Siberian Branch of Russian Academy of Sciences, Novosibirsk 630090, Russia

## Abstract

Using a recently developed approach to constructing covalent network structures from linear carbyne, we identify by *ab initio* calculations a new carbon allotrope in 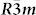 (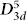) symmetry that comprises six-fold helical chains with alternating *sp*^2^-type single and double bonds along the chains that are connected via zigzag benzene rings. This 6-fold carbene is characterized as a three-dimensional three-connected chiral crystalline modification of graphite. Phonon and electronic band calculations indicate that this new structure is dynamically stable and is a semiconductor with a band gap of 0.47 eV, in contrast to the semimetallic nature of graphite. Simulated x-ray diffraction patterns of the 6-fold carbene provide an excellent match to the previously unexplained distinct diffraction peak of a new carbon allotrope found in recent detonation experiments. These results establish a new carbon phase and offer insights into its outstanding structural and electronic properties.

Carbon's extremely versatile bonding ability produces a rich variety of structural allotropes, among which graphite and diamond have long been known for their outstanding properties. All carbon atoms in graphite are connected via the *sp*^2^ hybridization bonding with a bond length of 0.142 nm and bond angle of 120°, and the graphite honeycomb lattice can be viewed as a planar molecule comprising benzene rings or 2-fold polyacetylene-like zigzag chains^1^. The strong in-plane aromatic *π*-conjugation makes the two-dimensional three-connected (2D3C) graphite the most stable allotropic form of carbon. In a diamond single crystal, all carbon atoms are connected via the *sp*^3^ hybridization bonding with the tetrahedral bond angle of 109.47° and a bond length of 0.154 nm. The average distance between its parallel planes is 0.140 nm, which is much smaller than that in graphite. Single crystal diamond has a dense 3D4C structure comprising four nearest-neighbor polyacetylene-like zigzag chains or two nearest-neighbor twisted graphite layers^2^. Under static pressure, the highly crystalline varieties of graphite can be transformed to diamond via slipping, buckling, and cross-linking of the carbon sheets^3^,^4^,^5^,^6^,^7^,^8^,^9^ with an *sp*^2^ → *sp*^3^ bonding transition. During the last two or three decades, tremendous attention has been focused on synthesizing a variety of new carbon allotropes, including 0D3C fullerenes^10^, 1D3C nanotubes^11^, and 2D3C graphene^12^ in all-*sp*^2^ networks. Novel fullerene structures have been discovered in natural coal^13^, rock^14^ and detonation soot^15^.

In addition to these well characterized carbon allotropes, there exist a number of proposed and synthesized carbon structures that require further exploration. Recently, we identified two 3D3C chiral framework structures in all-*sp*^2^ bonding networks comprising 3-fold and 4-fold helical chains connected by ethene-type (H_2_C = CH_2_) *π*-conjugation^16^. These structures contain matching helical chains of complementary chirality with ethene-type planar *π*-conjugation, and they are energetically more favorable than previously proposed structures with helical chains of same chirality (e.g., *K*_4_^17^) or zigzag chains (e.g., H-6^18^ and bct-4^19^) with twisted *π* bonds. This finding suggests a new approach to constructing covalent network structures. In particular, the matching helical chain configuration offers a new strategy for solving structures of yet unidentified carbon phases seen in recent experiments, including carbon blacks, soot, or similar materials^20^,^21^,^22^,^23^,^24^,^25^,^26^,^27^,^28^,^29^,^30^.

Here we report by *ab initio* total-energy and phonon calculations^31^,^32^,^33^,^34^,^35^ a new type of 3D3C crystalline carbon in an all-*sp*^2^ network that comprises 6-fold helical carbon chains with alternating single and double bonds, which are distinct from the polyyne-like alternating single and triple carbon-carbon bonds in linear *sp*-carbyne^36^. This structure can be regarded as twisted graphite in AA stacking consisting of zigzag hexagonal benzene rings connected by an ethene-type planar *π*-conjugation like as in the 3-fold and 4-fold carbene^16^. Phonon and electronic band calculations show that this new carbon phase is dynamically stable and is a semiconductor with a band gap of 0.47 eV, in contrast to the semimetallic nature of graphite. Simulated x-ray diffraction (XRD) patterns show an excellent match between this newly identified carbon structure and the condensed carbon phase discovered in detonation soot^28^,^29^,^30^. These results establish a new carbon phase and offer insights into its outstanding structural and electronic properties.

## Results

**Figure 1:**
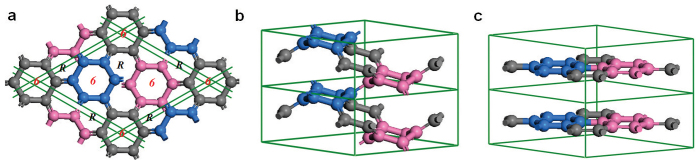
Chiral crystalline modification of carbon in an all-*sp*^2^ 3D3C bonding network in 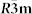 (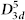) symmetry. Top (a) and side (b) view of rh6 carbon, which comprises three 6- fold helices or three zigzag benzene rings as its building blocks. In hexagonal representation its lattice parameters are *a* = 6.902 Å, *c* = 3.470 Å, and carbon atoms occupy the 18 h (0.8805, 0.1195, 0.5576) position. Symbols *R* and 6 represent right-handed helices and closed hexagon, respectively. It topologically corresponds to a (3 × 3 × 1) superlattice of graphite in AA stacking as shown in (c).

**Figure 2:**
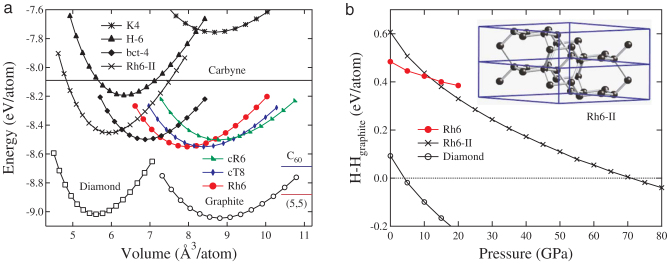
Energetics and kinetics of selected carbon structures. (a) Energy versus volume for *K*_4_, H-6, bct-4, cR6, cT8 and rh6 in 3D3C bonding networks compared to those of 1D2C carbyne, 2D3C graphite and 3D4C diamond. The energies for fcc C_60_ fullerene and (5,5) carbon nanotube are also shown for comparison. (b) Enthalpy per atom (relative to that of graphite) for rh6, rh6-II, and diamond versus pressure. The insert picture is rh6-II structure.

We first present structural characterization of the new carbon phase, which has a 6-atom rhombohedral primitive unit cell with equilibrium lattice parameters *a* = 4.1491 Å, *α* = 112.55°, occupying the 6 h (0.4381, 0.7965, 0.4381) position, thus termed rh6 carbon (or 6-fold carbene). In hexagonal representation, it has an 18 atom hexagonal unit cell (see Fig. 1a and 1b) with lattice parameters *a* = 6.902 Å, *c* = 3.470 Å, occupying the 18 h (0.8805, 0.1195, 0.5576) position. It has a polymerized carbyne-like structure containing three 6-fold helical carbon chains; however, instead of having the polyyne-like alternating *sp*-type single and triple carbon-carbon bonds as those in linear carbyne^36^, the right-handed (or left-handed) helical chains in rh6 carbon have an alternating *sp*^2^-type single and double carbon-carbon bonds (see Fig. 1a). The *sp* → *sp*^2^ bonding state transition produces a large energy gain of about 0.46 eV per carbon atom (see Fig. 2a). Meanwhile, three right-handed (or left-handed) helical carbon chains bond together forming a zigzag benzene ring. Therefore, rh6 carbon also can be regarded as a twisted graphite in AA stacking consisting of three zigzag hexagonal carbon rings with a (3 × 3 × 1) superlattice of graphite (see Fig. 1c). However, in contrast to the uniform bond length of 1.42 Å in graphite, there are two distinct carbon-carbon bond lengths, a longer bond of 1.483 Å associated with a C(*sp*^2^)–C(*sp*^2^) single bond in the zigzag benzene rings and a shorter bond of 1.359 Å associated with an ethene-type C(*sp*^2^) = C(*sp*^2^) double bond^16^ between the zigzag benzene rings (these bonds are different from that in 3-fold and 4-fold carbene with single bonds along the chains and double bonds between the carbon chains^16^). Also, there are two different bond angles, 113.45° for 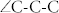 in the zigzag benzene rings, and 123.16° for 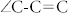 out of the zigzag benzene rings. These structural parameters on average are similar to those in graphite, but they are closer to those in 3-fold and 4-fold carbene^16^, thus rh6 carbon can also be considered a 6-fold chiral carbene.

We have calculated the total energy of rh6 carbon as a function of volume (see Fig. 2a) in comparison with the results for diamond and several other carbon structures that are all in an all-sp^2^ bonding environment, including *K*_4_^17^, H-6^18^, bct-4^19^, cR6^16^, cT8^16^, and graphite. The energies for fcc C_60_ fullerene^37^ and (5,5) carbon nanotube are also shown for comparison. Graphite has all its 2*p_z_* orbitals perfectly aligned, leading to a strong aromatic *π* bonding interaction between neighboring carbon atoms, which makes it the most stable carbon crystal. Conversely, all the 2*p_z_* orbitals are misaligned in the *K*_4_ carbon, making it the most unfavorable crystalline carbon form^16^. Meanwhile, rh6 carbon has one-third of its bonds with well-aligned 2*p_z_* orbitals between the zigzag benzene rings and, as a result, it is less stable than diamond, graphite, C_60_ fullerene, and (5,5) nanotube, but is almost as stable as the 4-fold cT8 carbene and more favorable than 3-fold cR6 carbene with the same 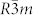 symmetry.

It should be noted that there is a dense form of rh6 (named rh6-II as shown in Fig. 2) with lattice parameters *a* = 7.012 Å, *c* = 2.509 Å, occupying the 18 h (0.7495, 0.1252, 0.4582) position. The rh6-II phase can be obtained from rh6 phase via local bond rotation (Fig. S1 in Supplementary Information) and stabilized under pressure above 12 GPa (see Fig. 2b). It is even more stable than graphite above 70 GPa due to the *sp*^3^-like bonding nature in rh6-II state. However, this structural transformation rh6 → rh6-II is reversible (Fig. S2 in Supplementary Information), and upon decompression rh6 phase is recovered. The calculated equilibrium structural parameters, total energy, bulk modulus, and electronic band gap for rh6, rh6-II, cR6 and cT8 are listed in Table I, compared to available experimental data^38^.

**Figure 3:**
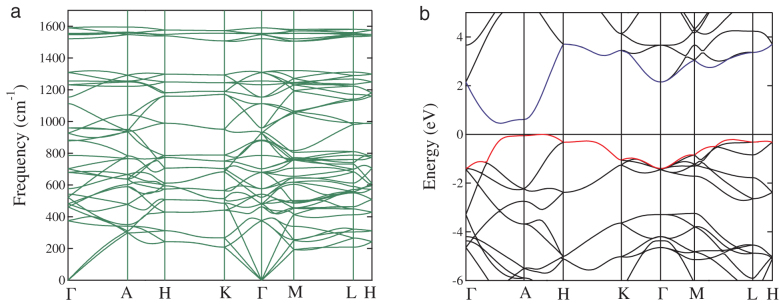
Phonon and electronic band structures. (a) Calculated phonon band structure of rh6 carbon at zero GPa. (b) Calculated electronic band structure of rh6 carbon at zero GPa.

To understand the mechanical stability of rh6 carbon, the corresponding elastic constants *C*_11_, *C*_33_, *C*_44_, *C*_12_, and *C*_13_ are estimated to be 630, 90, 128, 288, and 139 GPa, respectively. It clearly meets the mechanical stability criteria given by *C*_44_ > 0, *C*_11_ > |*C*_12_|, and 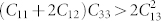 for hexagonal phase^39^. We further examined dynamic stability of rh6 carbon by calculating the phonon dispersion curves. No imaginary frequencies were observed throughout the entire Brillioun zone (results at 0 GPa are shown in Fig. 3a), confirming the dynamic stability of rh6 carbon. The highest phonon frequency 1611 cm^−1^ for rh6 carbon is almost equal to the highest phonon frequency of 1610 cm^−1^ for graphite^40^, which reflects the graphite-like structural bonding character of rh6 carbon.

The increased bonding connectivity in the 3D3C rh6 carbon is expected to open an electronic band gap in contrast to the semimetallic state in the 2D3C graphite structure. A hybrid density functional method based on the Heyd-Scuseria-Ernzerhof scheme (HSE06)^34^ has been used to calculate electronic properties. It is seen that the calculated HSE06 band gap (5.36 eV) for diamond is very close to the experimental data^38^ of 5.47 eV, suggesting the validity of HSE06 method in predicting band gaps for diamond. Our calculated electronic band structure of rh6 carbon (see Fig. 3b) indeed shows a band gap of 0.47 eV with the conduction band bottom and valence band top located along the Γ-A and A-H direction, respectively. This moderate band gap makes rh6 carbon highly desirable for a wide range of applications in electronic, optical, and energy conversion devices.

**Figure 4:**
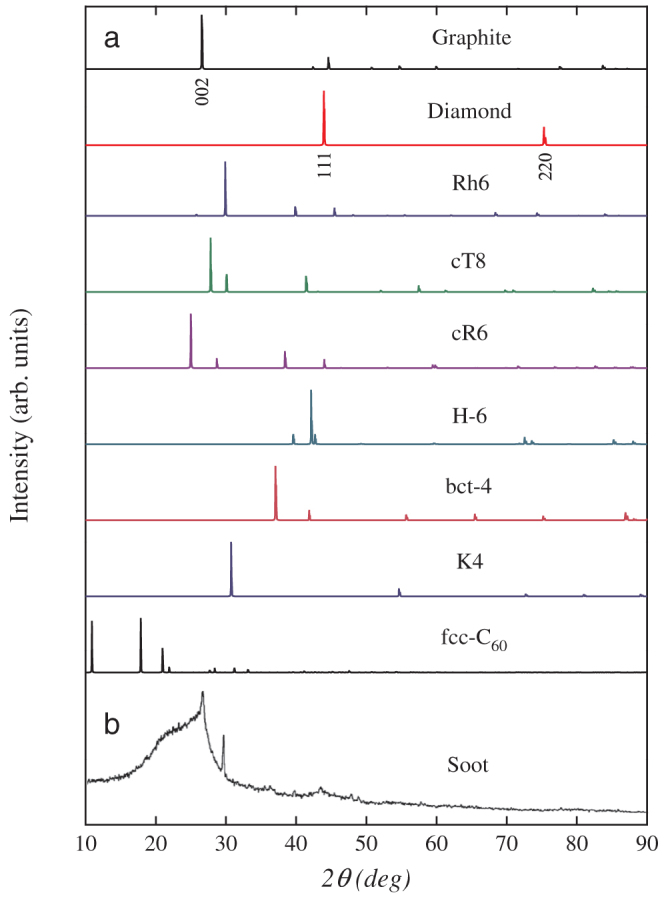
X-ray diffraction (XRD) patterns. (a) Simulated XRD patterns for graphite, diamond, rh6 carbon, and the other structures cT8, cR6, H-6, bct-4, *K*_4_, and fcc C_60_. (b) Experimental XRD patterns for TNT/diesel oil detonation soot^28^. X-ray wavelength is 1.5406 Å with a copper source.

We now establish the experimental connection of rh6 carbon. Several experiments in recent years have discovered a new carbon structure in detonation soot or similar materials^20^,^21^,^22^,^23^,^24^,^25^,^26^,^27^,^28^,^29^,^30^, but have not been able to identify its structural features that hold the key to further understanding and exploration of this new carbon phase. Experimental x-ray diffraction (XRD) of a TNT/dissel oil detonation soot^28^ (see Fig. 4b) revealed the presence of a considerable amount of amorphous carbon and several crystalline phases: the strongest peak around 26° comes from the graphite (002) diffraction; a weak peak around 43.6° is attributed to the diamond (111) diffraction; a prominent sharp diffraction peak at 30°, however, cannot be assigned to any known carbon phase such as graphite, diamond or fullerenes^25^,^26^,^27^. A similar sharp diffraction peak at 30° was also found in the chimney soot^29^ and the trinitrotoluene/cyclomethylenetrinitramine detonation nanoparticles^30^. The high intensity and sharpness of this unexplained XRD peak suggests that a new carbon phase has been consistently produced in these experiments. In Fig. 4a, we show simulated XRD patterns for graphite, diamond, rh6 carbon, cT8, cR6, H-6, bct-4, *K*_4_ and fcc C_60_ described in Fig. 2a, and compare with the experimental XRD pattern^28^ (see Fig. 4b). Our simulated XRD patterns for graphite and diamond are consistent with the corresponding experimental XRD peaks. Most importantly, the main peak for rh6 carbon matches almost perfectly the XRD peak at 30° for the newly identified carbon phase. Beside of rh6 carbon, it is shown that the main peak of 30.8° for *K*_4_ carbon is also close to 30°; however, the *K*_4_ carbon is dynamically unstable^41^. These results suggest that the new rh6 carbon is among the likely candidates for the unidentified carbon phases found in recent detonation experiments^28^.

It is well-known that Raman spectroscopy is also an appropriate method to study the characteristics of new phases of carbon. To provide more information and characters for possible experimental observation, we also simulated the Raman spectra of rh6 carbon and compared the results with different *sp*^2^ carbon structures (see Fig. S3 in Supplementary Information). We find that the Raman spectrum of rh6 carbon presents a main peak *A*_1*g*_ at 1605 cm^−1^ and a weaker shoulder peak *E_g_* at 1580 cm^−1^ instead of the *E*_2*g*_ mode at 1585 cm^−1^ in graphite^42^. These features may be helpful for identifying the new carbon phases in experiments^43^.

## Discussion

In summary, we have identified a new type of 3D3C crystalline carbon in an all-*sp*^2^ network in 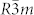 (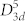) symmetry. It consists of three 6-fold carbon helices connected by three zigzag hexagonal carbon rings in hexagonal representation, and can be regarded as a chiral crystalline modification of graphite. Phonon and electronic band calculations indicate that this new carbon phase is dynamically stable and is a semiconductor with a band gap of 0.47 eV. Simulated XRD patterns provide an excellent match between this rh6 carbon phase and the previously unexplained carbon structure discovered in recent detonation experiments^28^,^29^,^30^. These results suggest that the possible presence of rh6 phase in soot carbon as well as the ultrafine diamond, graphite, and amorphous^28^.

Recently, a one-step, gas-phase, catalyst-free detonation of hydrocarbon (C_2_H_2_) method was developed to produce gram quantities of pristine graphene nanosheets^44^. It suggests an effective approach to synthesizing rh6 carbon via thermal decomposition or detonation of carbonaceous materials such as aromatic and polymeric hydrocarbon molecules. Successful production of rh6 carbon in large quantity is expected to lead to greatly increased research interest and activity, a situation reminiscent of the early days of the discovery and ensuing study of fullerenes.

## Methods

Calculations were carried out using the density functional theory as implemented in the Vienna *ab initio* simulation package (VASP)^31^. The generalized gradient approximation (GGA) developed by Armiento-Mattsson (AM05)^32^ were adopted for the exchange-correlation potential. The all-electron projector augmented wave (PAW) method^33^ was adopted with 2*s*^2^2*p*^2^ treated as valence electrons. A plane-wave basis set with a large energy cutoff of 800 eV was used. Forces on the ions are calculated through the Hellmann-Feynman theorem allowing a full geometry optimization. The energy minimization is done over the atomic and electronic degrees of freedom using the conjugate gradient iterative technique. Convergence criteria employed for both the electronic self-consistent relaxation and the ionic relaxation were set to 10^−8^ eV and 0.01 eV/Å for energy and force, respectively. A hybrid density functional method based on the Heyd-Scuseria-Ernzerhof scheme (HSE06)^34^ has been used to calculate electronic properties. Phonon dispersion curves and Raman spectrum are calculated using the package MedeA^35^ with the forces calculated from VASP. The phase conversion barrier was calculated using a generalized solid-state nudged elastic band method^45^,^46^ with the cell and atomic position optimization.

## Author Contributions

J.T.W., C.F.C. and E.G.W. designed the study and wrote the paper; J.T.W. and Y.K. carried out *ab initio* simulations; all authors discussed the results and contributed to the manuscript.

## Supplementary Material

Supplementary Information Supplementary information for "A New Carbon Allotrope with Six-Fold Helical Chains in all-sp2 Bonding Networks"

## Figures and Tables

**Table 1:** Calculated equilibrium structural parameters (space group, volume *V*_0_, lattice parameters *a* and *c*, bond lengths *d_C_*_–*C*_), total energy *E_tot_*, bulk modulus *B*_0_, and electronic band gap *E_g_* for cR6 carbon, cT8 carbon, rh6 carbon, graphite, and diamond at zero pressure, compared to available experimental data^38^

Structure	Method	*V*_0_(Å^3^/atom)	a (Å) c (Å)	*d_C–C_* (Å)	*E_tot_* (eV)	*B*_0_ (GPa)	*E_g_* (eV)
Diamond ( 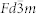 )	GGA	5.60	3.552	1.538	−9.018	451	5.36
	Exp^38^	5.67	3.567	1.544		446	5.47
cR6 ( 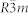 )	GGA	8.78	7.122 3.597	1.352, 1.490	−8.502	268	2.95
cT8 (*I*4_1_/*amd*)	GGA	8.40	5.937 3.808	1.351, 1.488	−8.551	283	2.41
Rh6 ( 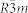 )	GGA	7.96	6.902 3.470	1.359, 1.483	−8.550	299	0.47
Rh6-II ( 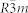 )	GGA	5.94	7.012 2.509	1.452, 1.536	−8.454	408	1.04
Graphite (*P*6_3_/*mmc*)	GGA	8.81	2.462 6.710	1.422	−9.045	280	
	Exp^38^	8.78	2.460 6.704	1.420		286	
