# Supplementary material for: A New Carbon Allotrope with Six-Fold Helical Chains in all-sp2 Bonding Networks
Source: Sci Rep. 2014 Mar 11;4:4339. doi: 10.1038/srep04339 (PMC3949244; doi:10.1038/srep04339)
Supplement: Supplementary Information — for "A New Carbon Allotrope with Six-Fold Helical Chains in all-sp2 Bonding Networks" [file srep04339-s1.doc]

**Supplementary information for “A New Carbon Allotrope with Six-Fold Helical Chains in all-sp^2^ Bonding Networks”**

Jian-Tao Wang^1,*^, Changfeng Chen^2^, Enge Wang^3^ & Yoshiyuki Kawazoe^4,5^

*^1^Beijing National Laboratory for Condensed Matter Physics, Institute of Physics, Chinese Academy of Sciences, Beijing 100190, China*

*^2^Department of Physics and High Pressure Science and Engineering Center, University of Nevada, Las Vegas, Nevada 89154, USA*

*^3^International Center for Quantum Materials, School of Physics, Peking University, Beijing 100871, China*

*^4^New Industry Creation Hatchery Center, Tohoku University, Sendai 980-8579, Japan*

*^5^Institute of Thermophysics, Siberian Branch of Russian Academy of Sciences, Novosibirsk 630090, Russia.*

*Correspondence and requests for materials should be addressed to J.T.W. [wjt@aphy.iphy.ac.cn].

1. **Kinetic stability of rh6 carbon**

To understand the kinetic stability of the newly identified rh6 carbon structure in all-sp^2^ bonding networks, we here examine the kinetic process at the atomic scale using a generalized solid-state nudged elastic band method^1–3^ with the cell and atomic position optimized under 0, 6, 12 GPa. For rh6 towards graphite (Fig. S1a), the conversion barrier at 0 GPa is estimated to be about 0.41 eV per atom (Fig. S2a), which is nearly identical to that for the diamond-to-graphite transition^1^. Consequently, rh6 carbon is expected to be as (meta)stable as diamond, thus highly viable at ambient conditions. Meanwhile, the conversion barrier from rh6 to rh6-II (Fig. S1b) is about 0.26 eV per atom at GPa (Fig. S2b). The phase conversion from rh6 to rh6-II always show smaller barrier in comparison with the phase conversion from rh6 towards graphite under pressure. These results suggest that the phase conversion from rh6 to rh6-II is more favorable than the phase conversion from rh6 to graphite under pressure. The rh6-II phase can be easy obtained from rh6 carbon via local bond rotation (Fig. S1b) with barrier of 0.04 eV per atom at 12 GPa, however, upon decompression rh6 phase is recovered from rh6-II phase with barrier of 0.13 eV per atom at 0 GPa. Therefore, the rh6 phase is more preferred by both kinetics and energetics at ambient conditions.


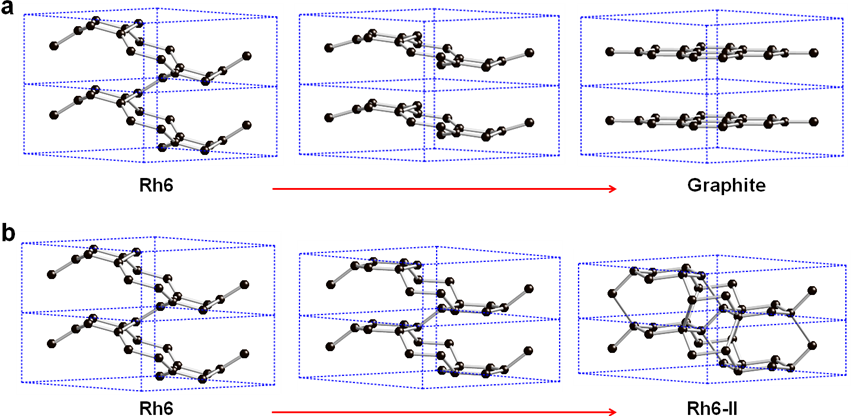


**Figure S1│Simulated local-bond-rotation reconstruction conversion processes.** (a) phase conversion from rh6 carbon toward graphite with local bond breaking at 6 GPa. (b) phase conversion from rh6 carbon toward rh6-II with local bond rotation at 6 GPa.

**Figure S2│Energy barrier curves along the structural evolution.** (a) Energy versus transformation pathway from rh6 towards graphite at 0, 6, 12 GPa. (b) Energy versus transformation pathway from rh6 towards rh6-II phase at 0, 6, 12 GPa.

**Figure S3│**Raman spectra for graphite, rh6, cT8 and cR6 carbon.

**2. Raman spectra of rh6 carbon**

To provide more information and characters for possible experimental observation, we also simulate the Raman spectra of rh6 carbon and compared the results with different sp^2^ carbon structures. The results are presented in Fig. S3. The *E_2g_* mode in graphite is estimated to be 1585 cm^-1^, which is well agreement with the experimental data^4^. Different from graphite, we find that the Raman spectrum of rh6 carbon presents a main peak *A_1g_* at 1605 cm^-1^ and a weaker shoulder peak *E_g_* at 1580 cm^-1^. These splitting peaks are corresponding to the *E_2g_* mode at 1585 cm^-1^ in graphite. Similar splitting features are also found in cT8 and cR6 carbon. These splitting features can be attributed to the bond change of aromatic pi-conjugation in graphite to ethene-type pi-conjugation in rh6 carbon as described in main text. Experimentally, one paper reported that the peak positions of G-band in carbon black are located at 1602-1608 cm^-1^ in contrast to 1582 cm^-1^ in graphite^5^. This shift is almost same as that in our rh6 and cR6 carbon (see Fig. S3). These features may be helpful for identifying the new carbon phases in experiments^5^.

References:

1. Wang, J. T., Chen, C. F. *&* Kawazoe, Y. Low-temperature phase transformation from graphite to *sp^3^* orthorhombic carbon. *Phys. Rev. Lett.* **106,** 075501 (2011).

2. Sheppard, D., Xiao, P., Chemelewski, W., Johnson, D. D. *&* Henkelman, G. A generalized solid-state nudged elastic band method. *J. Chem. Phys.* **136,** 074103 (2012).

3. Wang, J. T., Chen, C. F., Mizuseki, H. *&* Kawazoe, Y. Kinetic origin of divergent decompression pathways in silicon and germanium. *Phys. Rev. Lett.* **110,** 165503 (2013).

4. Ferrari, A. C. *et al.* Raman spectrum of graphene and graphene layers. *Phys. Rev. Lett.* **97,** 187401 (2006).

5. Jawhari, T., Roid, A. *&* Casado, J. Raman spectroscopy characterization of some commercially avalilable carbon black materials. *Carbon* **33,** 1561-1565 (1995).
